# Radiomics and Deep Features: Robust Classification of Brain Hemorrhages and Reproducibility Analysis Using a 3D Autoencoder Neural Network

**DOI:** 10.3390/bioengineering11070643

**Published:** 2024-06-24

**Authors:** Salar Bijari, Sahar Sayfollahi, Shiwa Mardokh-Rouhani, Sahar Bijari, Sadegh Moradian, Ziba Zahiri, Seyed Masoud Rezaeijo

**Affiliations:** 1 Department of Radiology, Faculty of Paramedical, Kurdistan University of Medical Sciences, Sanandaj P.O. Box 66177-13446, Iran; salar.bijari89@gmail.com; 2 Department of Neurosurgery, School of Medicine, Iran University of Medical Sciences, Tehran P.O. Box 14496-14535, Iran; sayfollahisahar@gmail.com; 3 Mechanical Engineering Group, Faculty of Engineering, University of Kurdistan, Sanandaj P.O. Box 66177-15175, Iran; mardokh94@gmail.com; 4 Department of Aging and Health, School of Public Health, Shahid Sadoughi University of Medical Sciences, Yazd P.O. Box 89151-73160, Iran; s.bijari8810@gmail.com; 5 Department of Radiology, Tehran University of Medical Sciences, Tehran P.O. Box 14197-33151, Iran; moradian.sm@gmail.com; 6 Department of Radiation Oncology, Golestan Hospital, Ahvaz Jundishapur University of Medical Sciences, Ahvaz P.O. Box 61357-15794, Iran; zibazaheir@gmail.com; 7 Department of Medical Physics, Faculty of Medicine, Ahvaz Jundishapur University of Medical Sciences, Ahvaz P.O. Box 61357-15794, Iran; 8 Cancer Research Center, Ahvaz Jundishapur University of Medical Sciences, Ahvaz P.O. Box 61357-15794, Iran

**Keywords:** reproducible, brain, hemorrhage, radiomics features, deep features, machine learning

## Abstract

This study evaluates the reproducibility of machine learning models that integrate radiomics and deep features (features extracted from a 3D autoencoder neural network) to classify various brain hemorrhages effectively. Using a dataset of 720 patients, we extracted 215 radiomics features (RFs) and 15,680 deep features (DFs) from CT brain images. With rigorous screening based on Intraclass Correlation Coefficient thresholds (>0.75), we identified 135 RFs and 1054 DFs for analysis. Feature selection techniques such as Boruta, Recursive Feature Elimination (RFE), XGBoost, and ExtraTreesClassifier were utilized alongside 11 classifiers, including AdaBoost, CatBoost, Decision Trees, LightGBM, Logistic Regression, Naive Bayes, Neural Networks, Random Forest, Support Vector Machines (SVM), and k-Nearest Neighbors (k-NN). Evaluation metrics included Area Under the Curve (AUC), Accuracy (ACC), Sensitivity (SEN), and F1-score. The model evaluation involved hyperparameter optimization, a 70:30 train–test split, and bootstrapping, further validated with the Wilcoxon signed-rank test and q-values. Notably, DFs showed higher accuracy. In the case of RFs, the Boruta + SVM combination emerged as the optimal model for AUC, ACC, and SEN, while XGBoost + Random Forest excelled in F1-score. Specifically, RFs achieved AUC, ACC, SEN, and F1-scores of 0.89, 0.85, 0.82, and 0.80, respectively. Among DFs, the ExtraTreesClassifier + Naive Bayes combination demonstrated remarkable performance, attaining an AUC of 0.96, ACC of 0.93, SEN of 0.92, and an F1-score of 0.92. Distinguished models in the RF category included SVM with Boruta, Logistic Regression with XGBoost, SVM with ExtraTreesClassifier, CatBoost with XGBoost, and Random Forest with XGBoost, each yielding significant q-values of 42. In the DFs realm, ExtraTreesClassifier + Naive Bayes, ExtraTreesClassifier + Random Forest, and Boruta + k-NN exhibited robustness, with 43, 43, and 41 significant q-values, respectively. This investigation underscores the potential of synergizing DFs with machine learning models to serve as valuable screening tools, thereby enhancing the interpretation of head CT scans for patients with brain hemorrhages.

## 1. Introduction

Brain hemorrhage is a critical and life-threatening medical condition that demands prompt and accurate evaluation and management. With a mortality rate of approximately 40%, early detection and precise classification of brain hemorrhage on non-contrast computed tomography (CT) scans are crucial for improving patient outcomes and minimizing neurological deficits [1,2,3]. However, the growing number of CT scans received by medical facilities can often lead to delays in diagnosis due to limited access to specialized radiologists, especially in academic institutions [4,5]. As a potential solution to address this challenge, an automatic notification system employing artificial intelligence (AI) methods has been proposed for efficient and timely brain hemorrhage detection [6,7,8,9,10].

Brain hemorrhage can result from various etiologies, including traumatic brain injury, hemorrhagic stroke, and subarachnoid hemorrhage caused by the rupture of intracranial aneurysms. These hemorrhages can cause increased intracerebral pressure and complications such as perihematomal edema or hydrocephalus, contributing to poor patient outcomes [11]. In a broader classification, brain hemorrhages can be categorized into two main types: traumatic and non-traumatic. Traumatic hemorrhages encompass extra-axial hemorrhages (e.g., subarachnoid hemorrhage (SAH), subdural hematoma (SDH), and epidural hematoma (EDH) and intra-axial hemorrhages (including cerebral contusion (CC)). Similarly, non-traumatic hemorrhages are further classified into extra-axial hemorrhages (e.g., subarachnoid hemorrhage (SAH)) and intraparenchymal hemorrhages (IPH), which involve lobar hematoma and centrally located hemorrhage [11,12].

An accurate and early distinction between different subtypes of brain hemorrhage is essential for appropriate patient management and treatment planning. However, visual inspection of brain CT images by human observers can be challenging and subject to interpretation variability. To overcome these limitations, radiomics and deep learning features have emerged as powerful quantitative imaging research methods. Radiomics allows for the non-invasive assessment of regional tissue heterogeneity at a millimeter scale, while deep learning techniques enable the extraction of complex and high-dimensional features from medical images. These advanced imaging approaches have shown promising applications in brain imaging and have the potential to revolutionize medical diagnosis and personalized treatment strategies [13,14,15,16].

In recent years, machine learning methods and radiomics analyses have been utilized to differentiate brain hemorrhage subtypes on CT images. This cutting-edge approach bridges the gap between conventional medical imaging practices and the emerging era of personalized medicine. Radiomics features (RF), extracted using various software packages, have been demonstrated to provide valuable insights into disease characterization and prediction. However, some RFs are sensitive to variations in image acquisition, reconstruction, and processing procedures, leading to potential inconsistencies and reduced reproducibility across studies. Ensuring robustness and reliability in feature extraction is essential to address these technical challenges and foster the widespread adoption of radiomics in clinical applications [17,18]. Moreover, while radiomics frameworks have shown promising results in predicting and diagnosing brain hemorrhage subtypes, the reproducibility of these results remains a paramount concern. Numerous studies have been conducted in this field; however, discrepancies in reported outcomes and a lack of standardized methodologies hinder the seamless translation of radiomics into clinical practice. Therefore, addressing the reproducibility of radiomics frameworks is a crucial step toward establishing this innovative imaging approach as a reliable and indispensable tool in the clinical setting [15,19,20,21,22,23,24].

The Intraclass Correlation Coefficient (ICC) was first introduced by Fisher in 1954 as a modification of the Pearson Correlation Coefficient. Currently, the ICC is determined using mean squares, which estimate population variances based on the variability within a given set of measurements, derived from analysis of variance. ICC is widely employed in conservative care medicine to evaluate interrater, test–retest, and intrarater reliability. Ensuring the reproducibility of radiomics, particularly the robustness of its measurements, is crucial for its application in clinical practice and medical research. The ICC, a widely accepted measure in various scientific fields, including radiology and medical imaging, offers several compelling advantages that justify its adoption. Historically, the ICC has been a reliable measure for assessing the agreement and consistency of measurements [23,24]. Its use is well documented in radiomics, where precision and reliability are paramount. Moreover, ICC’s capacity to account for various sources of variability, such as those from diverse imaging protocols, equipment, and patient populations, makes it a robust tool for radiomics reproducibility assessment [24,25,26,27,28,29,30,31]. This aligns with the overarching goal of radiomics, which is to consistently and accurately derive quantitative information from medical images. Furthermore, ICC’s adaptability and versatility make it a valuable asset in radiomics research [25]. It transcends the constraints of measurement scales and data types, enabling its application to a wide range of radiomic features and datasets [26,28,30,31,32,33,34]. This adaptability is invaluable in handling the inherent diversity of radiomics data. Additionally, ICC produces interpretable results that facilitate meaningful comparisons and inference. Researchers and practitioners in the field are adept at interpreting ICC values, which allows for effective communication of the reliability of radiomics measurements. Overall, the ICC coefficient effectively reduces errors stemming from individual variability or the extraction methodology.

In light of these considerations, this study aims to investigate the identification of robust radiomics and deep features extracted from a 3D autoencoder neural network (DFs) in CT brain imaging using the ICC. By employing the ICC metric, we aim to quantify the agreement and consistency of feature extraction across image samples. Furthermore, we intend to apply selected RFs and DFs (with ICC values exceeding 0.75) extracted from each CT image to various state-of-the-art classifiers for the accurate prediction and classification of brain hemorrhage subtypes. To the best of our knowledge, this study represents the first comprehensive investigation into the reproducibility of RFs and DFs in CT imaging for classifying brain hemorrhages into six subtypes (SAH, EDH, CC, SAH, IPH, and IVH).

## 2. Materials and Methods

### 2.1. Research Participant Selection and CT Imaging Approach

**Figure 1:**
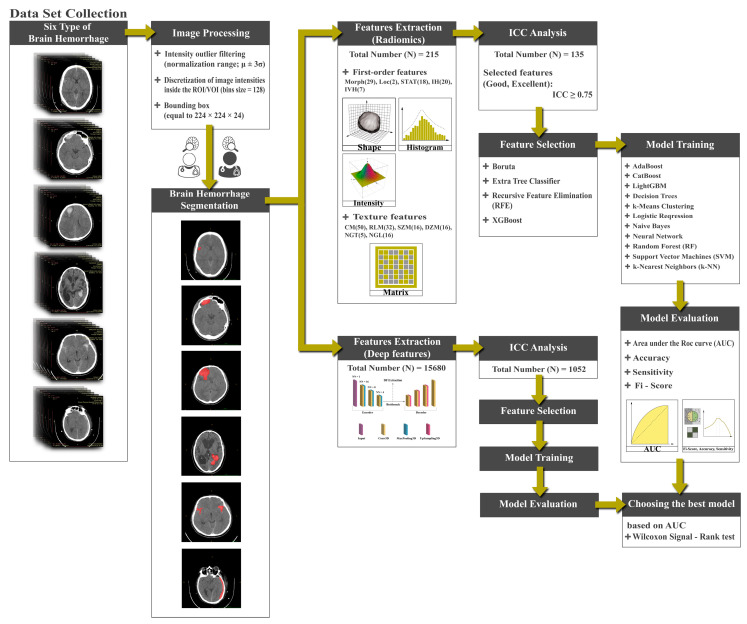
Flowchart of the proposed approach.

A general diagram showing the major steps of the proposed methodology is presented in Figure 1. The experimental procedures undertaken in this retrospective study received approval from the institutional committee of Ahvaz Jondishapur University of Medical Sciences (AJUMS) University. It is worth noting that our study was conducted retrospectively, and the AJUMS University institutional committee waived the requirement for informed consent. All methods were carried out following relevant guidelines and regulations.

Through a meticulous and systematic exploration of our institutional data repository, our goal was to identify patients who exhibited either non-traumatic or traumatic cerebral hemorrhage, as evident in their CT scans. To ensure the consistency and reliability of our results, we established a set of exclusion criteria. These included (1) the presence of multiple hemorrhagic lesions, which might introduce complexities in the interpretation of results; (2) inadequate imaging quality or improper imaging settings, which could compromise the accuracy of our study; (3) undetermined origin of the ailment, since the certainty of etiology is crucial in developing a precise diagnostic model; and (4) hematomas detected at fewer or equal to three layers, as our focus is primarily on substantial intracranial hemorrhages.

The development of our AI diagnostic support models necessitated the use of a comprehensive and varied dataset. Hence, we integrated data from an array of patients presenting different types of hemorrhages. The distribution of the patient data, according to the type of hemorrhage, was carefully selected to reflect the varied occurrence rates in the general population, a critical factor in developing a broadly applicable diagnostic tool. The dataset included 120 patients showing signs of subdural hemorrhage (SDH) represented by 3840 slices (16.67%), 60 patients with epidural hematoma (EDH) represented by 1920 slices (8.33%), 180 patients with cerebral contusions (CC) represented by 5760 slices (25%), 120 patients with subarachnoid hemorrhage (SAH) represented by 3864 slices (16.67%), 150 patients with intraparenchymal hemorrhage (IPH) represented by 4804 slices (20.83%), and finally, 90 patients without intraventricular hemorrhage (IVH) represented by 2887 slices (12.5%). The dataset was meticulously compiled from four different institutions, each contributing imaging data acquired over a period spanning from August 2016 to December 2022. This deliberate inclusion of data from multiple centers ensures a substantial temporal spread and variability in patient populations, imaging protocols, and equipment. The choice of a multicenter dataset is motivated by the recognition that the reproducibility of radiomics and deep learning techniques is a critical aspect of their clinical utility. Imaging was conducted using a 64-channel CT scanner (LightSpeed VCT, GE Healthcare), with parameters meticulously selected to ensure high-quality scans. These included detector collimation set at 64 × 0.625 mm, rotation time of 0.5 s, pitch of 1.375, and tube voltage at 120 kV. The tube current was modulated between 250 and 400 mA. Of the total dataset employed for the development (720 patients), a significant proportion (690 patients or 95.83%) possessed a slice thickness of 5 mm, with the remaining slices varying between 3.0 mm and 10 mm.

### 2.2. Segmentation of Intracranial Hemorrhage

**Figure 2:**
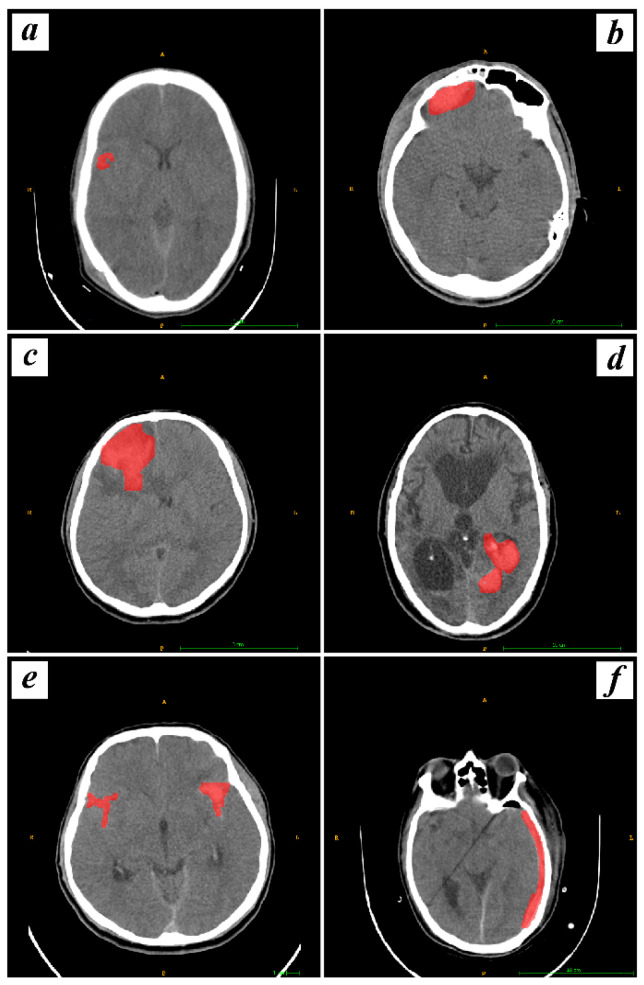
Visualization of manual segmentation for hemorrhagic types, including CC, EDH, IPH, IVH, SAH, and SDH, labeled as (**a**–**f**) respectively, with hemorrhages marked in red.

In pursuit of determining the volume of interest (VOI) for subsequent analysis, we delineated all the contiguous slices of the entire hemorrhage. Furthermore, our inclusion criteria specified that lesions should be visible in at least three slices. This criterion ensures that the radiomics and deep analysis are conducted on lesions with adequate spatial representation, aligning with the three-dimensional nature of our imaging data. Limiting our analysis to substantial intracranial hemorrhages observed in three or more layers enhances the robustness and reliability of our radiomics features extraction methodology. Contours were sketched marginally within the periphery of the hemorrhagic masses for SDH, EDH, CC, SAH, IPH, and IVH. These VOIs were delineated by two experienced medical professionals, with Reader 1 having ten years of experience in detecting hemorrhage in CT images and Reader 2 boasting five years of similar experience. Importantly, this segmentation process was repeated twice weekly to ensure that the same radiomic features were calculated at least two times, meeting the criteria for ICC calculation. The segmentation, conducted independently by Reader 1 and Reader 2, was performed with diligence and adherence to established protocols. This rigorous approach involved the assessment of the same set of images on two separate occasions during each week, allowing for the calculation of ICC to evaluate intraobserver reliability. The rationale behind involving two experienced radiologists was to enhance the robustness of the segmentation process and minimize potential bias. Both physicians were not privy to the clinical and pathological information of the patients. An illustration of this manual segmentation process is provided in Figure 2.

### 2.3. Radiomics and Deep Features (Features Extracted from a 3D Autoencoder) Extraction

This research endeavor employed a comprehensive methodology for the extraction of features, encompassing both radiomics-based features (RFs) and DFs. The RFs were obtained utilizing the standardized Software Environment for Radiomic Analysis (SERA), a framework renowned for its robustness and effectiveness in feature extraction from medical images. Additionally, DFs were extracted through a 3D autoencoder.

In the radiomics feature extraction phase, a total of 215 quantitative RFs were extracted from CT images utilizing SERA. Among these features, 79 were categorized as first-order features, and the remaining 136 features comprised 3D features, including 29 morphology features (Morph), 2 local intensity features (LOC), 18 statistics features (STAT), 24 intensity histogram features (IH), 7 intensity volume histogram features (IVH), 50 co-occurrence matrix (3D, averaged and merged) features (CM), 32 run length matrix (3D, averaged and merged) features (RLM), 16 size zone matrix (3D) features (SZM), 16 distance zone matrix (3D) features (DZM), 5 neighborhood gray tone difference matrix (3D) features (NGT), and 16 neighboring gray level dependence matrix (3D) features (NGL).

**Figure 3:**
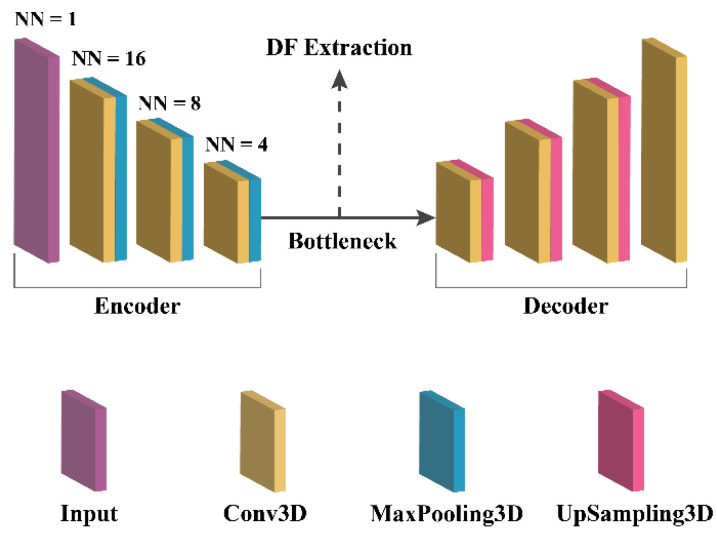
3D autoencoder architecture.

To extract deep features (DFs), a 3D autoencoder neural network architecture was meticulously implemented. An autoencoder consists of an encoder network, which maps input images to a latent representation or bottleneck, and a decoder network, which reconstructs the original images from the latent representation, as shown in Figure 3. For this study, the encoder network adhered to a standard convolutional architecture, consisting of three 3 × 3 convolutional layers, each followed by a leaky rectified linear unit (LeakyReLU) activation and a 2 × 2 max-pooling operation for parameter reduction. The decoder path comprised three 3 × 3 convolutional layers, each followed by a LeakyReLU activation and an up-sampling operation.

Training the proposed autoencoder involved the following steps and parameters:

- Loss Function: The binary cross-entropy loss function was minimized to train the autoencoder.
- Optimization Algorithm: The Adam optimization algorithm was used for its adaptive learning rate capabilities.
- Learning Rate: The learning rate was carefully set to 0.001 to balance convergence speed and optimization stability.
- Number of Epochs: The training process spanned 20 epochs.
- Batch Size: A batch size of 8 was employed during training.
- Dataset: The dataset utilized for training and validation included all available 3D CT images, ensuring comprehensive representation. The data were randomly split into training and testing sets with a 70:30 ratio. Finally, the bootstrapping technique was utilized.
- Feature Extraction: A total of 15,680 features were extracted from the bottleneck layer of the 3D autoencoder model.

### 2.4. Assessment of Reproducibility, Feature Selection, and Classification

The reproducibility of the DFs and RFs was evaluated using the Intraclass Correlation Coefficient (ICC) with carefully selected parameters. These parameters included two-way random effects, absolute agreement, and multiple raters/measurements. The ICC is a widely used reliability index for measuring agreement between continuous variables in reproducible studies. It is a ratio ranging from 0 to 1. Based on the ICC value, reliability can be categorized as Poor (ICC < 0.5), Moderate (0.5 ≤ ICC < 0.75), Good (0.75 ≤ ICC < 0.9), or Excellent (ICC ≥ 0.9). Features with an ICC value of 0.75 or greater were considered for further analysis. To compute the ICC, an in-house-developed Python code was utilized. Additionally, Pearson’s Correlation Coefficient was employed to identify redundant features (correlation coefficient > 0.9). Hence, we computed the average of features with high reliability and non-redundancy for further analysis. Next, we utilized Boruta, Recursive Feature Elimination (RFE), and XGBoost techniques, along with the ExtraTreesClassifier, to identify the most relevant features. Subsequently, we employed 11 classifiers: AdaBoost, CatBoost, Decision Trees, K-Means Clustering, LightGBM, Logistic Regression, Naive Bayes, Neural Networks, Random Forest, Support Vector Machines (SVM), and k-Nearest Neighbors (k-NN) for the classification of Intracranial Hemorrhage. Next, we conducted hyperparameter optimization using a train–test split approach with a ratio of 70:30. The grid search technique was applied to explore the optimal hyperparameters on the training dataset. This process aims to fine-tune the model’s performance by systematically searching through a predefined hyperparameter space and identifying the combination that yields the best results. Subsequently, we executed 1000 bootstraps with a ratio of 0.9 on the test dataset. This comprehensive approach resulted in a total of 44 models for each of the six brain hemorrhage types, comprising four feature selection methods and eleven distinct machine learning models. The evaluation of these models was carried out using four well-established metrics, namely, Area Under the Curve (AUC), Accuracy (ACC), Sensitivity (SEN), and F1-score. The AUC values for all the models were subjected to a comparative analysis using the Wilcoxon signed-rank test, alongside the application of q-values to control for multiple testing. Multiple testing issues arise when conducting numerous statistical tests simultaneously, leading to an increased risk of obtaining false positives. To address this concern, we employed q-values as a robust tool in comparing different machine learning models. Q-values offer a controlled approach to the false discovery rate (FDR) and play a pivotal role in mitigating the impact of multiple comparisons. The method involves assigning a q-value to each observed *p*-value, representing the minimum FDR at which the corresponding test may be deemed significant. By utilizing q-values, we ensure a more stringent control over false positives, enabling a more accurate assessment of observed differences in model performance. This approach is particularly relevant in our study, where the comparison of machine learning models necessitates careful consideration of the potential for inflated significance levels due to multiple testing. A threshold of 0.05 was set to identify statistically significant results. The implementation of all the models and analyses was performed in R programming language version 4.0, utilizing the mlr library version 2.18 on the Ubuntu 18.04 operating system.

## 3. Results

### 3.1. Demographic Characteristics and Imaging Finding

The demographic characteristics and CT findings of brain hemorrhage patients are presented in Table 1. The study encompassed 120 patients with SDH, 60 patients with EDH, 180 patients with CC, 120 patients with SAH, 150 patients with IPH, and 90 patients with IVH. The statistical analysis of these parameters was performed to investigate any potential associations. Regarding age, the mean (±standard deviation) age for SDH, EDH, CC, SAH, IPH, and IVH patients were 40 ± 28 years, 45 ± 18 years, 47 ± 19 years, 46 ± 21 years, 45 ± 17 years, and 48 ± 16 years, respectively. The Kruskal–Wallis chi-squared analysis indicated no significant difference in age distribution among the different types of brain hemorrhage (*p*-value = 0.775). With respect to gender distribution, the number of male patients was 71 (59.2%), 32 (53.3%), 92 (51.1%), 68 (56.7%), 80 (53.3%), and 70 (77.8%) in the SDH, EDH, CC, SAH, IPH, and IVH groups, respectively. However, the Multinomial Chi-square test did not reveal a statistically significant difference in gender distribution across the various types of hemorrhage (*p*-value = 0.351). The analysis of drug medication usage showed that 8 (6.7%), 4 (6.7%), 11 (6.1%), 9 (7.5%), 2 (1.3%), and 61 (67.8%) patients in the SDH, EDH, CC, SAH, IPH, and IVH groups, respectively, had a history of drug medication. Nevertheless, the association between drug medication and brain hemorrhage did not reach statistical significance (*p*-value = 0.302). The medical history of the patients revealed varying patterns of underlying conditions. Notably, 4 (3.3%) SDH patients had a history of high blood pressure, whereas in the EDH, CC, SAH, IPH, and IVH groups, the number of patients with high blood pressure were 0 (0%), 4 (2.2%), 6 (5%), 60 (40%), and 45 (50%), respectively. Arteriovenous malformations (AVMs) were observed in 1 (0.8%) CC patient, 1 (1.7%) SAH patient, 7 (4.7%) IPH patients, and 4 (4.4%) IVH patients. Additionally, cerebral aneurysms were reported in 2 (1.3%) IPH patients and 2 (2.2%) IVH patients. Tumor cases were observed in 4 (3.3%) SDH patients, 1 (1.7%) EDH patient, 3 (1.7%) CC patients, 2 (1.7%) SAH patients, 18 (12%) IPH patients, and 2 (2.2%) IVH patients. However, further analysis indicated that these medical history associations were not statistically significant (*p*-value = 0.334).

### 3.2. Analyzing Radiomics Features and Classifiers Performance

#### 3.2.1. Analyzing RFs Based on ICC

In this section, we explore the reliability and robustness of RFs and categorize these features based on their ICC. The primary objective is to assess the ICC as a measure of reliability for our RFs. To achieve this, we manually classified the RFs into four distinct categories based on their ICC values. Table 2 presents a comprehensive breakdown of the various features associated with each reliability category. The rows of the table are labeled as Poor, Moderate, Good, and Excellent, each representing a specific reliability category. The columns, on the other hand, indicate the feature categories under examination.

I. Poor Reliability Category:

This category comprises 43 features. Among the different feature categories, we observe that RLM has the highest representation with 9 features. Following closely, CM, MORPH, STAT, IH, and SZM have 8, 8, 5, 4, and 3 features, respectively. Other features like DZM, NGL, NGT, and IVH also contribute with 2, 2, 1, and 1 features, respectively. However, some feature categories like LOC do not have any occurrences in this reliability category.

II. Moderate Reliability Category:

This category encompasses 37 features. Here, the distribution of features is unbalanced similar to the Poor category. RLM and IH remain prominent, with 11 and 6 features, respectively. Additionally, MORPH, CM, and SZM each have 4 features. STAT, DZM, and NGL have representation in this category, with 3, 2, and 2 features, respectively. However, some feature categories like NGT and LOC do not have any occurrences in this reliability category.

III. Good Reliability Category:

The Good category consists of 45 features. In this category, CM retains its significance with 8 features. Other features like IH, MORPH, STAT, and DZM are represented with 7, 7, 5, and 5 features, respectively.

IV. Excellent Reliability Category:

The Excellent category comprises 90 features. Here, we observe that CM stands out with a substantial presence of 30 features. MORPH, NGL, and RLM also remain significant with 10, 10, and 8 features, respectively, and DZM, IH, SZM, and STAT each have 7, 7, 6, and 5 features. Other features like LOC, IH, IVH, and NGT have limited representation.

Overall, the analysis presented in Table 2 allows us to gain valuable insights into the distribution and reliability of RFs across various feature categories. By categorizing the RFs based on ICC, we have provided a comprehensive overview of their performance, thereby facilitating a better understanding of their robustness and applicability for our study.

**Figure 4:**
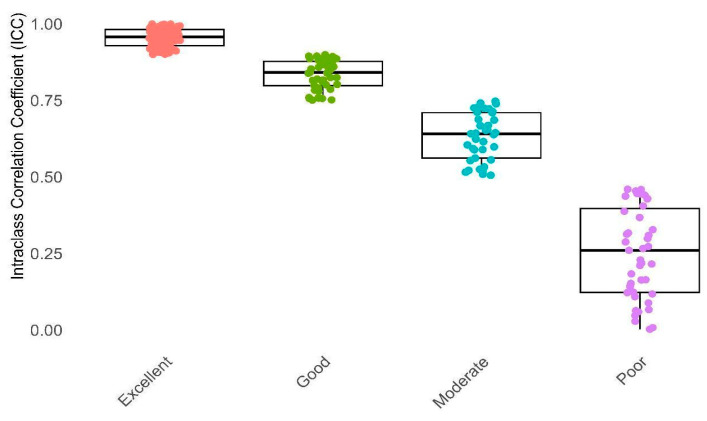
This box plot illustrates Intraclass Correlation Coefficient (ICC) values categorized into four reliability groups: Poor, Moderate, Good, and Excellent. The X-axis distinguishes between these reliability categories, while the Y-axis represents the ICC values. Reliability categorization: Poor (ICC < 0.5), Moderate (0.5 ≤ ICC < 0.75), Good (0.75 ≤ ICC < 0.9), Excellent (ICC ≥ 0.9).

**Figure 5:**
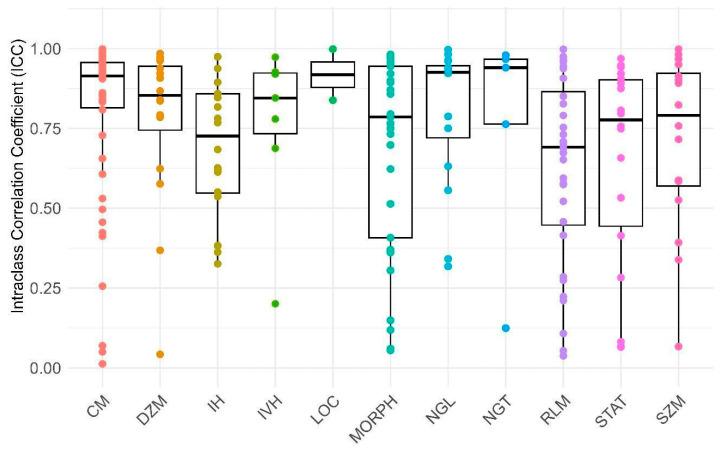
This box plot displays the Intraclass Correlation Coefficient (ICC) values for various reliability factors (RFs) related to every group of features. The X-axis represents the specific RFs, while the Y-axis indicates the corresponding ICC values. The abbreviations used are as follows: MORPH: morphology, LOC: location, STAT: statistic, IH: intensity histogram, IVH: intensity-volume histogram, CM: co-occurrence matrix, RLM: run length matrix, SZM: size zone matrix, DZM: distance zone matrix, NGT: neighboring gray tone, NGL: neighboring gray level.

Furthermore, Figure 4 and Figure 5 illustrate distinct distributions of features related to the defined reliability and feature categories in comparison to their ICC values, respectively. As depicted in Figure 4 and Figure 5 and Table 2, it can be observed that 41.86% of the features, particularly those pertaining to NGL, NGT, and CM, fall within the excellent reliability category. Additionally, 20.94% of the features, notably DZM, LOC, and IH, exhibit good reliability. Meanwhile, 17.2% of the features, especially RLM, SZM, and IH, demonstrate moderate reliability. In contrast, a subgroup of features amounting to 20%, particularly those encompassing RLM, STAT, and MORPH, is categorized under poor reliability. In general, 62.79% of all features had a suitable ICC (good and excellent) and others excluded from study.

**Figure 6:**
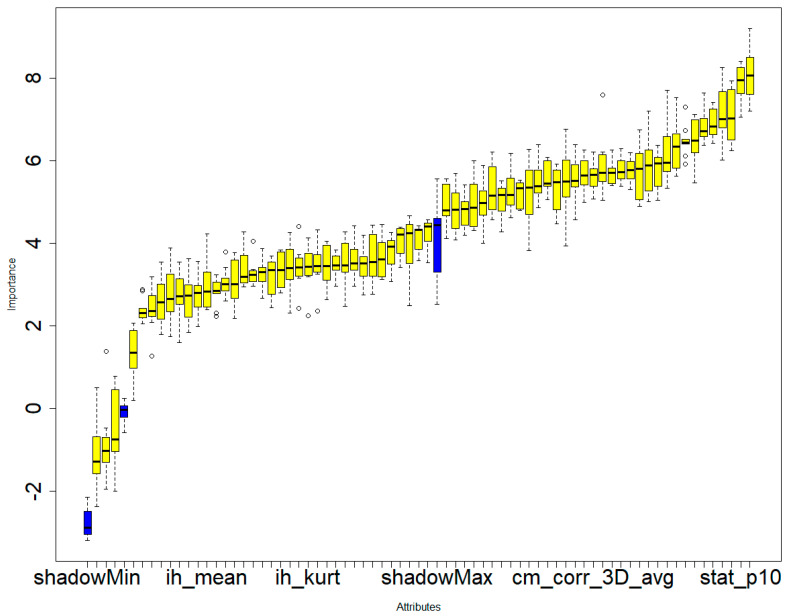
Boruta’s feature selection method illustrating the significance of various features in the differential diagnosis of six types of brain hemorrhages: SDH, EDH, CC, SAH, IPH, and IVH. The three blue boxes represent the minimal, average, and maximal importance of the shadow attributes.Only confirmed features for which the importance was significantly larger than that of the shadow variables were chosen as the final selected features for constructing the radiomics model. A total of 69 radiomics features were selected to construct the radiomics model.

**Figure 7:**
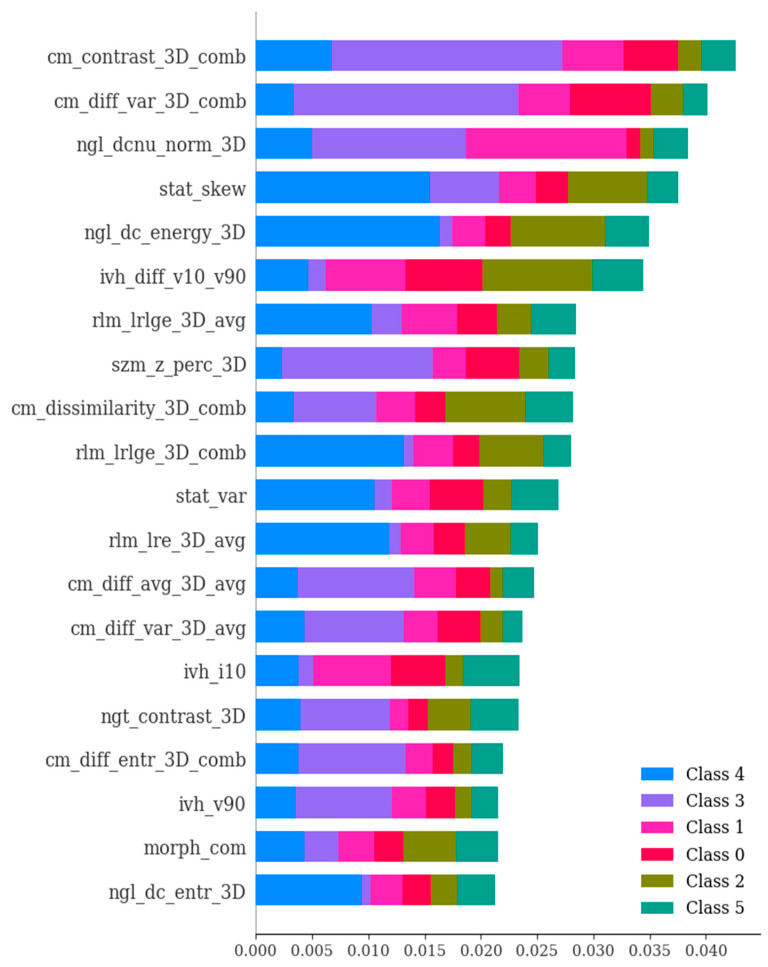
The ExtraTreesClassifier method showcasing the significance of various features in the differential diagnosis of six types of brain hemorrhage, namely, SDH, EDH, CC, SAH, IPH, and IVH. The ExtraTreesClassifier represents an ensemble tree-based machine learning approach that leverages randomization to mitigate variance and computational expenses, as compared to the Random Forest method. The importance values of features were ranked with respect to their role in the differential diagnosis of the six brain hemorrhage types. The figure highlights the most crucial features, which have been selected based on SHAP analysis.

**Figure 8:**
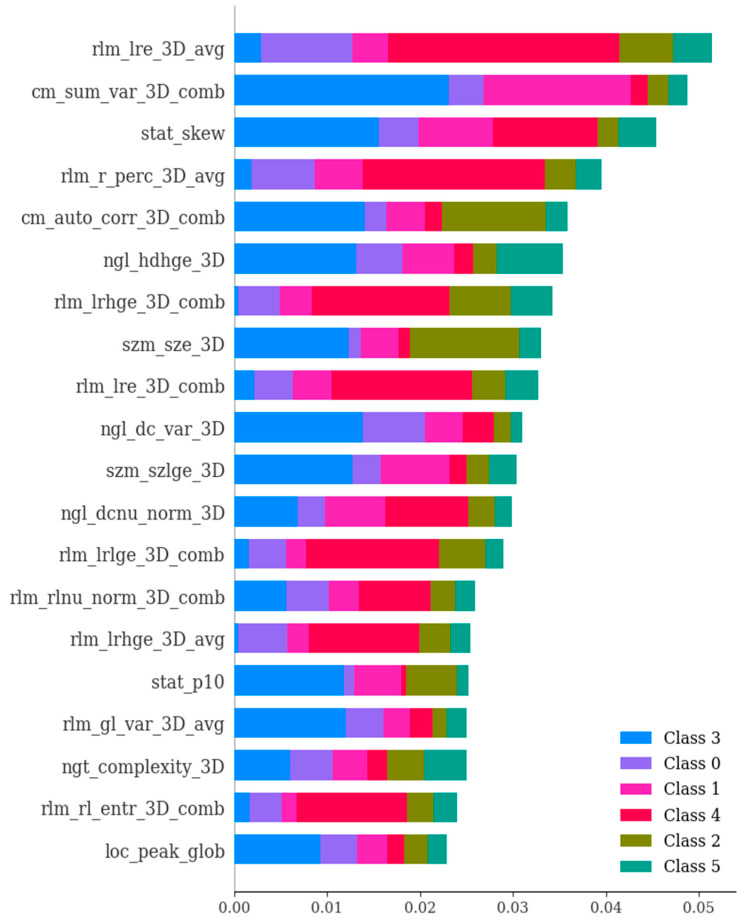
The RFE technique showcasing the significance of various features in the differential diagnosis of six types of brain hemorrhages, namely, subdural hematoma (SDH), epidural hematoma (EDH), cerebral contusion (CC), subarachnoid hemorrhage (SAH), intraventricular hemorrhage (IVH), and intraparenchymal hemorrhage (IPH). The selection process systematically eliminates less relevant features one by one until reaching the optimal number required for peak performance. The features were ranked based on their importance values in the context of differentiating among the six types of brain hemorrhages. The figure highlights the most crucial features selected using the SHAP (SHapley Additive exPlanations) method.

**Figure 9:**
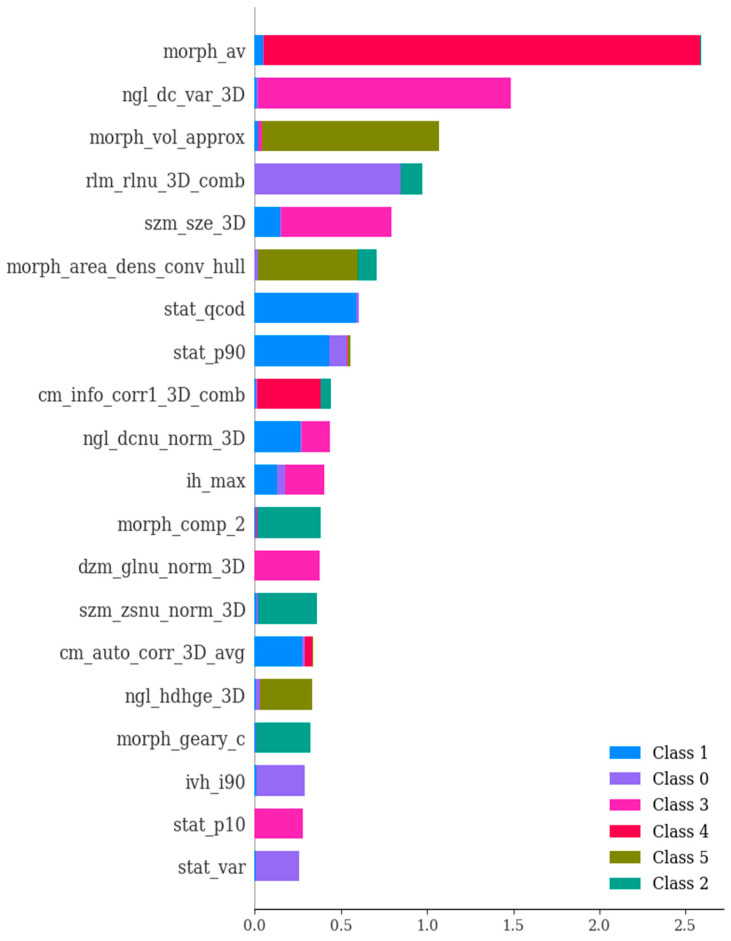
The importance of various features in the differential diagnosis of six types of brain hemorrhage, namely, SDH, EDH, CC, SAH, IPH, and IVH. In essence, this depiction showcases the scores that signify the utility and value of each individual feature during the creation of the boosted decision trees within the model. The ranking of features is determined based on their importance values concerning the differentiation of the six brain hemorrhage types. Notably, the figure presents the most crucial features selected through the utilization of SHAP (SHapley Additive exPlanations).

In the second phase of our study, we embarked on an exploration involving 135 dependable RFs, all of which demonstrated an ICC surpassing 0.75. These 135 features exhibited ICC values that exceeded 0.75, signifying a strong level of agreement both between different observers and within the same observer for these specific attributes. Employing a univariate correlation analysis, we unearthed that 112 radiomics features displayed significant variations across different types of brain hemorrhagic conditions. Subsequently, these features underwent a sequential assessment using Boruta, ExtraTreesClassifier, RFE, and XGBoost methodologies. This comprehensive approach led to the identification of the most crucial features (as depicted in Figure 6, Figure 7, Figure 8 and Figure 9).

#### 3.2.2. Classifiers Performance for RFs

**Figure 10:**
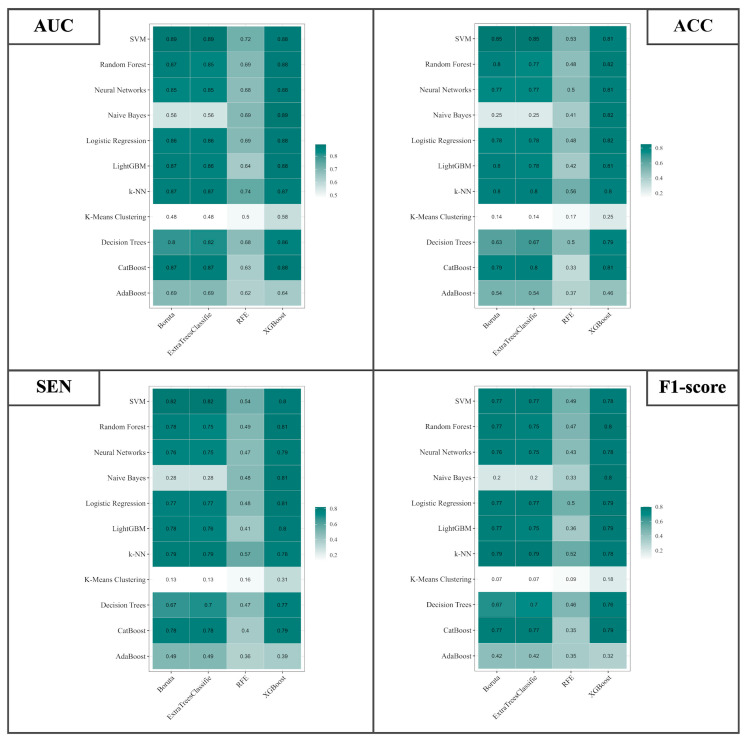
Illustration of the AUC, ACC, SEN, and F1-Score for differential diagnosis of SDH, EDH, CC, SAH, IPH, and IVH using 4 different feature selection methods and 11 classifiers based on RFs.

The current study delves into the critical domain of differential diagnosis encompassing SDH, EDH, CC, SAH, IPH, and IVH. The investigation entails a meticulous evaluation employing an ensemble of eleven classifiers coupled with four distinct feature selection methodologies. The metrics under scrutiny include the AUC, ACC, SEN, and F1-Score. The visual representations in Figure 10 encapsulate the overarching performance metrics. It is elucidated that the best-performing classifier–feature selection combinations are unveiled for each of the metrics. For AUC, the combination of SVM with Boruta feature selection and SVM with ExtraTreesClassifier achieved an AUC value of 0.89. Similarly, for ACC, SVM with Boruta and SVM with ExtraTreesClassifier reached an ACC of 0.85. The metric of SEN was maximized at 0.82, through the deployment of SVM with Boruta and SVM with ExtraTreesClassifier. However, for F1-Score, the combination of Random Forest + XGBoost and Naive Bayes + XGBoost achieved an F1-Score of 0.8. Moreover, the results encompass several noteworthy alternatives. Notably, for AUC, commendable outcomes were attained with SVM combined with XGBoost, Random Forest combined with XGBoost, Neural Networks combined with XGBoost, and Logistic Regression combined with XGBoost, all exhibiting an AUC of 0.88. The pivotal role of XGBoost in feature selection for AUC is evident from the results. In congruence, ACC exhibited promising results through Random Forest combined with XGBoost, Naive Bayes combined with XGBoost, and Logistic Regression combined with XGBoost, all registering an ACC of 0.82. Here again, XGBoost demonstrated its efficacy in feature selection. SEN values mirrored these trends, with Random Forest combined with XGBoost, Naive Bayes combined with XGBoost, and Logistic Regression combined with XGBoost yielding a sensitivity of 0.81. Subsequently, F1-Score indicated favorable outcomes through Logistic Regression combined with XGBoost, LightGBM combined with XGBoost, KNN combined with ExtraTreesClassifier, and KNN combined with Boruta, all culminating in an F1-Score of 0.79.

### 3.3. Analyzing Deep Features and Classifiers Performance

**Figure 11:**
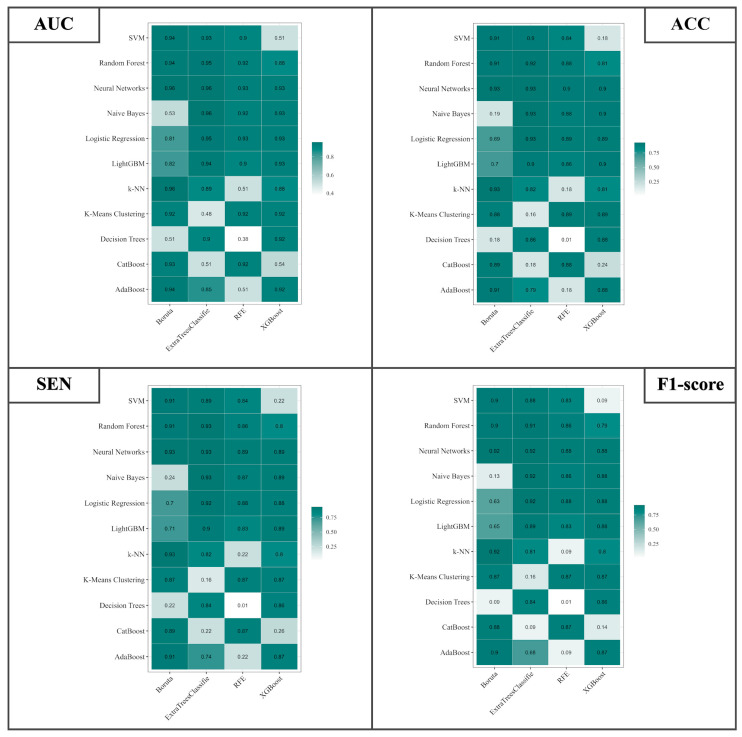
Illustration of the AUC, ACC, SEN, and F1-Score for differential diagnosis of SDH, EDH, CC, SAH, IPH, and IVH using 4 different feature selection methods and 11 classifiers based on DFs.

A comprehensive collection of 15,680 deep attributes was systematically derived from each brain CT scan image through the utilization of the 3D CNN. Notably, a subset of 1000 attributes exhibited ICCs surpassing the threshold of 0.75, signifying commendable levels of inter-observer consensus for these specific attributes. Employing a univariate correlation analysis, the deep attributes showcased noteworthy distinctions across various classifications of brain hemorrhagic conditions. In harmony with the approach taken for radiomics attributes, graphical representations serve as illuminating depictions of performance metrics associated with the differentiation of SDH, EDH, CC, SAH, IPH, and IVH. The focal point of this visual narrative is encapsulated within Figure 11, encapsulating the holistic performance metrics of the diagnostic endeavor. What is revealed is that the best combinations of classifier and feature selection methods have been identified for each specific measure. Specifically, in terms of the AUC, combining Naive Bayes with ExtraTreesClassifier feature selection and Neural Networks with ExtraTreesClassifier resulted in a high AUC of 0.96. Similarly, when considering ACC, notable results were achieved by combining Naive Bayes with ExtraTreesClassifier, Logistic Regression with ExtraTreesClassifier, and Neural Networks with ExtraTreesClassifier, leading to an ACC of 0.93. The highest SEN was achieved at 0.93 through a strategic approach involving Naive Bayes with ExtraTreesClassifier, Logistic Regression with ExtraTreesClassifier, Neural Networks with ExtraTreesClassifier, and Neural Networks with Boruta. Likewise, the F1-Score metric reached a peak of 0.92, facilitated by coordinating Naive Bayes with ExtraTreesClassifier, Logistic Regression with ExtraTreesClassifier, Neural Networks with ExtraTreesClassifier, and Neural Networks with Boruta.

### 3.4. Wilcoxon Signed-Rank Test for RFs and DFs

**Figure 12:**
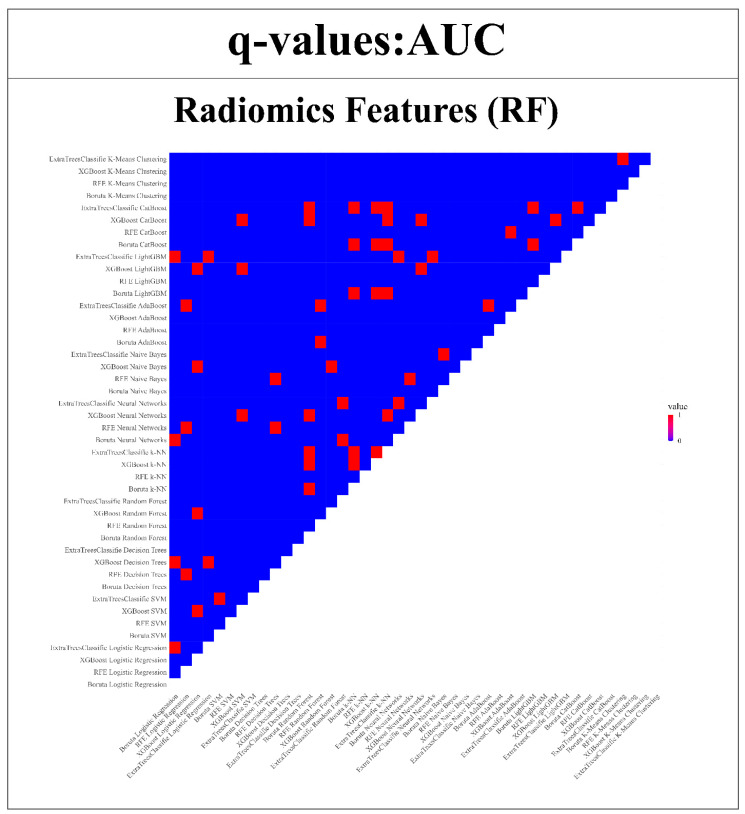
Wilcoxon signed-rank test is used to compare the AUC of RFs models compared with all other 43 models. (Q-value > 0.05 = red, q-value ≤ 0.05 = blue).

**Figure 13:**
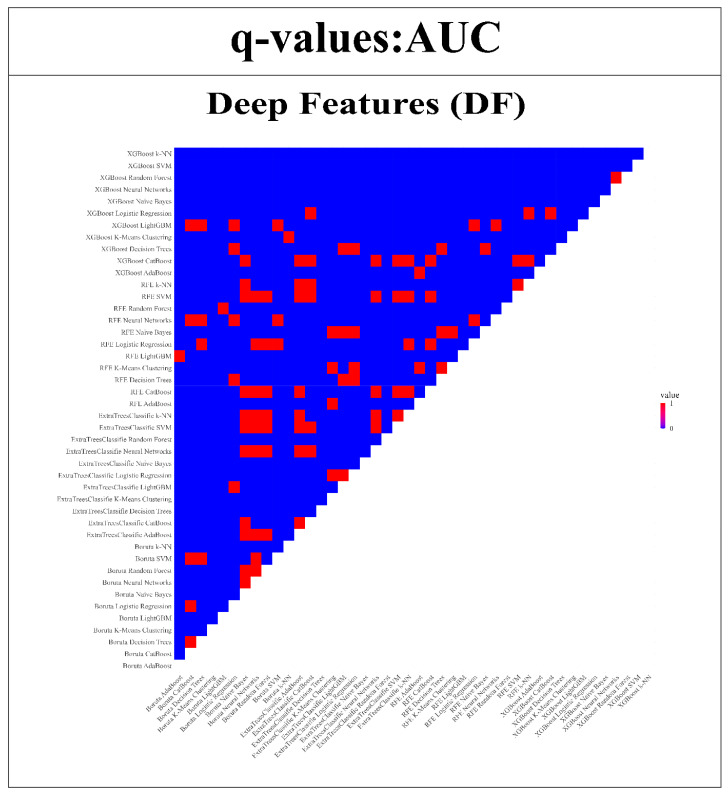
Wilcoxon signed-rank test is used to compare the AUC of DFs models compared with all other 43 models. (Q-value > 0.05 = red, q-value ≤ 0.05 = blue).

The strength of the proposed models was further validated by subjecting them to the Wilcoxon signed-rank test. This statistical analysis enabled a thorough comparison of model performance across each of the endpoints in relation to the remaining 43 models. The insights obtained from this examination are depicted in Figure 12 and Figure 13. The uniqueness of the models is highlighted through color-coded annotations indicating significant q-values. Among the RFs, noteworthy performers include SVM with Boruta, Logistic Regression with XGBoost, SVM with Extra TreesClassifier, CatBoost with XGBoost, and Random Forest with XGBoost. These models exhibited 42, 42, 41, 41, and 41 significant q-values, respectively, showcasing their robust discriminatory capabilities. However, in the case of DFs, remarkable performers comprise ExtraTreesClassifier + Naive Bayes, ExtraTreesClassifier + Random Forest, and Boruta + k-NN, each with 43, 43, and 41 significant q-values.

## 4. Discussion

This study employed a comprehensive and systematic approach to tackle the challenge of differentially diagnosing various types of brain hemorrhage. This was achieved through the utilization of RFs and DFs. The research methodology encompassed several key steps, including participant selection, CT imaging protocols, segmentation of intracranial hemorrhage, extraction of features, and classification using a diverse range of machine learning techniques. An essential focal point of the study involved evaluating the reliability of both RFs and DFs. The ICC was employed to categorize features based on their reliability, shedding light on their distribution across distinct categories. Notably, approximately 41.86% of these features exhibited excellent reliability, underscoring their potential importance within the diagnostic framework. To identify the most pertinent features for classification, various feature selection techniques were applied, including Boruta, ExtraTreesClassifier, RFE, and XGBoost. The classification phase encompassed a variety of classifiers, and the ensuing performance metrics (AUC, ACC, SEN, and F1-Score) underscored the effectiveness of different classifier feature selection combinations. In a similar vein, DFs extracted using a 3D autoencoder underwent analogous classification procedures. The reliability of these DFs demonstrated parity with radiomics-based features and further highlighted their significance in the context of differentiating brain hemorrhage types. The fusion of classifiers and feature selection methodologies yielded impressive performance metrics, thereby showcasing the potential efficacy of deep features in achieving accurate classification. The corroborative Wilcoxon signed-rank test reinforced the robustness of the models, revealing substantial variations in model performance and bolstering the credibility of both radiomics-based and deep feature-based approaches. The general review (35–43) of other articles is shown in Table 3.

Matsoukas et al.’s [8] systematic review brought together a compilation of studies involving AI algorithms for ICH detection and CMBs on medical images. Their comprehensive analysis revealed pooled estimates of AI tool performance, demonstrating remarkable sensitivity, specificity, and accuracy. Their results underscore the potential of AI-driven solutions to significantly enhance the diagnostic process for ICH and CMBs. While our study specifically investigates RFs and DFs reliability and classifier performance, our results parallel their findings by showcasing the high potential for AI-driven models to excel in diagnosing brain hemorrhage conditions. Seyam et al.’s [32] research focused on implementing an AI-based detection tool for ICH in a real-time clinical workflow. They demonstrated both the diagnostic accuracy and the potential workflow improvements that AI tools can offer. Although they encountered challenges in detecting specific subtypes of ICH, their work emphasizes the practical integration of AI algorithms into real-world clinical settings. Our study, in comparison, provides a multicenter dataset of patients with brain hemorrhage, the reliability of radiomics and deep features, and the performance of various classifiers. While our focus differs, the overarching theme of AI’s potential to enhance clinical practice aligns with Seyam et al.’s findings. Lee et al.’s [33] work introduced a deep-learning algorithm for ICH detection, presenting an alternative approach to conventional CNNs. Their study emphasized diagnostic performance and its practical implications, highlighting the algorithm’s potential to expedite diagnosis in emergency situations. While Lee et al.’s [33] work showcases an algorithmic approach, our study complements this by demonstrating the broader spectrum of analysis possible in the field of AI and medicine. In the study conducted by Angkurawaranon et al. [34], the focus lies on the critical realm of ICH stemming from traumatic brain injury (TBI). Their investigation underscores the urgency of timely radiological assessment and physician recognition in such scenarios. The advent of CT scanning, chosen as the investigation method of preference for TBI cases, gains prominence due to the scarcity of trained radiology personnel. Against this backdrop, Angkurawaranon et al. [34] delved into the potential of deep learning models as a promising avenue to expedite the generation of accurate radiology reports. Their study not only evaluates the diagnostic proficiency of a deep learning model but also contrasts its performance with the abilities of radiology, emergency medicine, and neurosurgery residents in detecting, localizing, and classifying traumatic ICHs. Remarkably, the deep learning model achieved a commendable level of accuracy at 0.89, surpassing the residents in sensitivity at 0.82. However, it is noteworthy that the specificity of 0.90 demonstrated room for improvement. Overall, the research highlights the potential utility of the deep learning model as a screening tool to support the interpretation of head CT scans in the context of traumatic brain injury patients. In comparing these findings with our study, both studies collectively emphasize the substantial promise of AI-driven tools in enhancing diagnostic accuracy and clinical decision-making for various brain hemorrhage conditions.

In the context of these studies, our research further expands the understanding of AI’s potential in diagnosing brain hemorrhage conditions. By delving into various machine learning techniques and using RFs and DFs reliability, we provide a more holistic perspective on AI’s applications. The robustness of our proposed models, as indicated by the Wilcoxon signed-rank test, adds to the mounting evidence of AI’s proficiency in medical diagnosis. Collectively, our study’s results converge with the findings of other authors, supporting the notion that AI-driven tools hold substantial promise in enhancing diagnostic accuracy, clinical workflows, and emergency care in the domain of brain hemorrhage detection. Our research contributes a distinct analytical dimension, which, when juxtaposed with these existing studies, portrays a more comprehensive landscape of AI’s role in advancing medical practice. Our study advocates for a judicious approach, emphasizing the need for rigorous validation studies that span different clinical settings. These studies are crucial not only for gaining confidence in the robustness of radiomics and deep learning techniques but also for fostering their adaptability across a spectrum of real-world scenarios.

## 5. Conclusions

Our study primarily focused on assessing the reproducibility of a machine learning model incorporating RFs and DFs for the effective classification of various types of brain hemorrhages. The ICC was employed as a metric to gauge the agreement and consistency of feature extraction, aligning with the study’s second aim. Specifically, our meticulous screening process, guided by ICC thresholds (>0.75), led to the identification of 135 RFs and 1054 DFs deemed robust for further analysis. This step directly addresses the aim to quantify the agreement and consistency of feature extraction across image samples, demonstrating our commitment to methodological rigor. In conclusion, the study’s aims were systematically integrated into the research methodology, and the results presented in the conclusion section succinctly encapsulate the outcomes achieved in the context of investigating robust radiomics and deep features using the ICC. We believe that our approach not only aligns with the stated aims but also contributes valuable insights to the field of machine learning in the context of brain hemorrhage classification.

The outcomes of this study not only contribute to enhancing the accuracy of brain hemorrhage classification but also shed light on the intricate interplay between demographic characteristics, imaging features, and advanced artificial intelligence techniques in the medical domain. This research serves as a crucial stepping stone toward improved clinical decision-making and patient care, as well as a potential framework for further advancements in the fusion of artificial intelligence and medical diagnostics.

## Figures and Tables

**Table 1:** Comparison of demographic features and CT findings among various types of brain hemorrhage patients.

Demographic Features	SDH (120)	EDH (60)	CC (180)	SAH (120)	IPH (150)	IVH (90)	*p*-Value
Age (years, mean (mean ± SD))	40 ± 28	45 ± 18	47 ± 19	46 ± 21	45 ± 17	48 ± 16	0775
Sex male, n (%)	71	32	92	68	80	70	0.351
Drug medication	8	4	11	9	2	61	0.302
Medical history							0334
High blood pressure	4	0	4	6	60	45	
Arteriovenous malformations (AVMs)	0	0	1	1	7	4	
Cerebral aneurysms	0	0	0	0	2	2	
Tumor	4	1	3	2	18	2	

**Table 2:** Distribution of features by reliability categories in RF analysis.

	Poor	Moderate	Good	Excellent	Total
MORPH	8	4	7	10	29
LOC	0	0	1	1	2
STAT	5	3	5	5	18
IH	4	6	7	7	24
IVH	1	1	2	3	7
CM	8	4	8	30	50
RLM	9	11	4	8	32
SZM	3	4	3	6	16
DZM	2	2	5	7	16
NGT	1	0	1	3	5
NGL	2	2	2	10	16
Total	43	37	45	90	215

**Table 3:** Comparing the proposed method for the differential diagnosis of cerebral hemorrhages with other developed approaches.

Names	Index Test	Training Labels	Model Outputs	Train Groups	Test Groups	AUC	Sensi-tivity	Speci-ficity
Chang et al. (2018) [35]	CNN mask R-CNN	Manual segmentation	Binary prediction of ICH	10,159	682	0.981	0951	0.973
Salehinejad et al. (2021) [36]	CNN ensemble: ResNeXt-50 and ResNeXt-101	Slice-level binary presence of abnormality (present/not present)	Binary prediction of ICH	21,784	5965	0.954	0.912	0.941
Prevedello et al. (2017) [37]	CNN GoogLe-Net	Examination-level presence of abnormality (present/not present)	Binary prediction of pathology (present/not present)	197	80	0.91	0.900	0.850
Chilamkurthy et al. (2018) [38]	Qure.ai proprie-tary CNN ResNet18	Slice-level binary presence of abnormality (present/not present)	Binary prediction of ICH (present/not present)	290,055	491	0.962	0.949	0.865
Arbabshirani et al. (2018) [39]	CNN	Examination-level binary presence of abnormality (present/not present)	Binary prediction of ICH (present/not present) for each examination	24,882	347	-	0.698	0.871
Ginat et al. (2020) [40]	Aidoc		Binary prediction of ICH	-	8723	-	0.884	0.961
Monteiro et al. (2020) [41]	CNN, Deep-Medic	Semi automatically created segmentations	Binary prediction of ICH (present/not present)	655		0.83	0.898	0.509
Kuo et al. (2019) [42]	CNN, ‘Patch-FCN’ (modified ResNet-38	Manual segmentations	Binary prediction of ICH (present/not present)	4396	200	0.991	1.00	0.87
McLouth et al. (2021) [43]	Avi-cenna.ai, CINA v1.0	-	Binary prediction of acute, hyperdense ICH (present/not present)	8994	814	-	0.914	0.975
Our study	RFs RFs DFs	Manual segmentations	Classifying brain hemorrhages into six subtypes (SAH, EDH, CC, SAH, IPH, and IVH)	504	216	0.89 0.96	0.82 0.92	- -

## Data Availability

The data are not publicly available due to their containing information that could compromise the privacy of research participants. The source code or scripts that were used to generate results will be released soon at the following link: https://github.com/MASOUD-AJUMS/Reproducible-Brain-Hemorrhage-Classification-via-Radiomics-and-Deep-Features.git_.

## References

[1] Little J.R., Dial B., Bélanger G., Carpenter S. (1979). Brain hemorrhage from intracranial tumor. Stroke.

[2] Hanley D.F. (2009). Intraventricular hemorrhage: Severity factor and treatment target in spontaneous intracerebral hemorrhage. Stroke..

[3] Weisberg L.A. (1990). How to identify and manage brain hemorrhage. Postgrad. Med..

[4] Kidwell C.S., Chalela J., Saver J.L., Hill M.D., Demchuk A., Butman J., Warach S. (2004). Comparison of MRI and CT for detection of acute intracerebral hemorrhage. JAMA.

[5] Heit J.J., Iv M., Wintermark M. (2017). Imaging of intracranial hemorrhage. J. Stroke.

[6] Rao B., Zohrabian V., Cedeno P., Saha A., Pahade J., Davis M.A. (2021). Utility of artificial intelligence tool as a prospective radiology peer reviewer—Detection of unreported intracranial hemorrhage. Acad. Radiol..

[7] Chan T. (2007). Computer aided detection of small acute intracranial hemorrhage on computer tomography of brain. Comput. Med. Imaging Graph..

[8] Matsoukas S., Scaggiante J., Schuldt B.R., Smith C.J., Chennareddy S., Kalagara R., Majidi S., Bederson J.B., Fifi J.T., Mocco J. (2022). Accuracy of artificial intelligence for the detection of intracranial hemorrhage and chronic cerebral microbleeds: A systematic review and pooled analysis. La Radiol. Medica.

[9] Parizel P., Makkat S., Van Miert E., Van Goethem J., Van den Hauwe L., De Schepper A. (2001). Intracranial hemorrhage: Principles of CT and MRI interpretation. Eur. Radiol..

[10] Zhang G., Chen K., Xu S., Cho P.C., Nan Y., Zhou X., Lv C., Li C., Xie G. (2021). Lesion synthesis to improve intracranial hemorrhage detection and classification for CT images. Comput. Med. Imaging Graph..

[11] Tan A.P., Svrckova P., Cowan F., Chong W.K., Mankad K. (2018). Intracranial hemorrhage in neonates: A review of etiologies, patterns and predicted clinical outcomes. Eur. J. Paediatr. Neurol..

[12] Ikram M.A., Wieberdink R.G., Koudstaal P.J. (2012). International epidemiology of intracerebral hemorrhage. Curr. Atheroscler. Rep..

[13] Fadavi P., Bagherzadeh S., Torabinezhad F., Goli-Ahmadabad F., Beiki M., Bijari S., Sayfollahi S., Momeni Z. (2023). Long-term study of vocal dysfunction and quality of life in patients with non-laryngeal head and neck cancers post chemo-radiation therapy: Results of prospective analysis. Int. J. Radiat. Res..

[14] Rezaeijo S.M., Chegeni N., Baghaei Naeini F., Makris D., Bakas S. (2023). Within-modality synthesis and novel radiomic evaluation of brain MRI scans. Cancers.

[15] Fatan M., Hosseinzadeh M., Askari D., Sheikhi H., Rezaeijo S.M., Salmanpour M.R. (2021). Fusion-based head and neck tumor segmentation and survival prediction using robust deep learning techniques and advanced hybrid machine learning systems. 3D Head and Neck Tumor Segmentation in PET/CT Challenge.

[16] Pham C.H., Tor-Díez C., Meunier H., Bednarek N., Fablet R., Passat N., Rousseau F. (2019). Multiscale brain MRI super-resolution using deep 3D convolutional networks. Comput. Med. Imaging Graph..

[17] Bijari S., Jahanbakhshi A., Hajishafiezahramini P., Abdolmaleki P. (2022). Differentiating glioblastoma multiforme from brain metastases using multidimensional radiomics features derived from MRI and multiple machine learning models. BioMed Res. Int..

[18] Bijari S., Jahanbakhshi A., Abdolmaleki P. (2023). Non-invasive radiomics nomogram model for determining the low and high-grade glioma base on MRI images. Int. J. Radiat. Res..

[19] Whybra P., Zwanenburg A., Andrearczyk V., Schaer R., Apte A.P., Ayotte A., Baheti B., Bakas S., Bettinelli A., Boellaard R. (2024). The image biomarker standardization initiative: Standardized convolutional filters for reproducible radiomics and enhanced clinical insights. Radiology.

[20] Salmanpour M.R., Hosseinzadeh M., Akbari A., Borazjani K., Mojallal K., Askari D., Hajianfar G., Rezaeijo S.M., Ghaemi M.M., Nabizadeh A.H. Prediction of TNM stage in head and neck cancer using hybrid machine learning systems and radiomics features. Proceedings of the Medical Imaging 2022: Computer-Aided Diagnosis.

[21] Hosseinzadeh M., Gorji A., Fathi Jouzdani A., Rezaeijo S.M., Rahmim A., Salmanpour M.R. (2023). Prediction of Cognitive Decline in Parkinson’s Disease Using Clinical and DAT SPECT Imaging Features, and Hybrid Machine Learning Systems. Diagnostics.

[22] Heydarheydari S., Birgani M.J., Rezaeijo S.M. (2023). Auto-segmentation of head and neck tumors in positron emission tomography images using non-local means and morphological frameworks. Pol. J. Radiol..

[23] Shahzadi I., Zwanenburg A., Lattermann A., Linge A., Baldus C., Peeken J.C., Combs S.E., Diefenhardt M., Rödel C., Kirste S. (2022). Analysis of MRI and CT-based radiomics features for personalized treatment in locally advanced rectal cancer and external validation of published radiomics models. Sci. Rep..

[24] Xue C., Yuan J., Lo G.G., Chang A.T., Poon D.M., Wong O.L., Zhou Y., Chu W.C. (2021). Radiomics feature reliability assessed by intraclass correlation coefficient: A systematic review. Quant. Imaging Med. Surg..

[25] Fiset S., Welch M.L., Weiss J., Pintilie M., Conway J.L., Milosevic M., Fyles A., Traverso A., Jaffray D., Metser U. (2019). Repeatability and reproducibility of MRI-based radiomic features in cervical cancer. Radiother. Oncol..

[26] Pavic M., Bogowicz M., Würms X., Glatz S., Finazzi T., Riesterer O., Roesch J., Rudofsky L., Friess M., Veit-Haibach P. (2018). Influence of inter-observer delineation variability on radiomics stability in different tumor sites. Acta Oncol..

[27] Xue C., Yuan J., Poon D.M., Zhou Y., Yang B., Yu S.K., Cheung Y.K. (2021). Reliability of MRI radiomics features in MR-guided radiotherapy for prostate cancer: Repeatability, reproducibility, and within-subject agreement. Med. Phys..

[28] Yip S.S.F., Aerts H.J.W.L. (2016). Applications and limitations of radiomics. Phys. Med. Biol..

[29] Avanzo M., Wei L., Stancanello J., Vallières M., Rao A., Morin O., Mattonen S.A., El Naqa I. (2020). Machine and deep learning methods for radiomics. Med. Phys..

[30] Zhao B. (2021). Understanding sources of variation to improve the reproducibility of radiomics. Front. Oncol..

[31] Park J.E., Park S.Y., Kim H.J., Kim H.S. (2019). Reproducibility and generalizability in radiomics modeling: Possible strategies in radiologic and statistical perspectives. Korean J. Radiol..

[32] Seyam M., Weikert T., Sauter A., Brehm A., Psychogios M.-N., Blackham K.A. (2022). Utilization of artificial intelligence–based intracranial hemorrhage detection on emergent noncontrast CT images in clinical workflow. Radiol. Artif. Intell..

[33] Lee J.Y., Kim J.S., Kim T.Y., Kim Y.S. (2020). Detection and classification of intracranial haemorrhage on CT images using a novel deep-learning algorithm. Sci. Rep..

[34] Angkurawaranon S., Sanorsieng N., Unsrisong K., Inkeaw P., Sripan P., Khumrin P., Angkurawaranon C., Vaniyapong T., Chitapanarux I. (2023). A comparison of performance between a deep learning model with residents for localization and classification of intracranial hemorrhage. Sci. Rep..

[35] Chang P.D., Kuoy E., Grinband J., Weinberg B.D., Thompson M., Homo R., Chen J., Abcede H., Shafie M., Sugrue L. (2018). Hybrid 3D/2D convolutional neural network for hemorrhage evaluation on head CT. Am. J. Neuroradiol..

[36] Salehinejad H., Kitamura J., Ditkofsky N., Lin A., Bharatha A., Suthiphosuwan S., Lin H.M., Wilson J.R., Mamdani M., Colak E. (2021). A real-world demonstration of machine learning generalizability in the detection of intracranial hemorrhage on head computerized tomography. Sci. Rep..

[37] Prevedello L.M., Erdal B.S., Ryu J.L., Little K.J., Demirer M., Qian S., White R.D. (2017). Automated critical test findings identification and online notification system using artificial intelligence in imaging. Radiology.

[38] Chilamkurthy S., Ghosh R., Tanamala S., Biviji M., Campeau N.G., Venugopal V.K., Mahajan V., Rao P., Warier P. (2018). Deep learning algorithms for detection of critical findings in head CT scans: A retrospective study. Lancet.

[39] Arbabshirani M.R., Fornwalt B.K., Mongelluzzo G.J., Suever J.D., Geise B.D., Patel A.A., Moore G.J. (2018). Advanced machine learning in action: Identification of intracranial hemorrhage on computed tomography scans of the head with clinical workflow integration. NPJ Digit. Med..

[40] Ginat D. (2021). Implementation of machine learning software on the radiology worklist decreases scan view delay for the detection of intracranial hemorrhage on CT. Brain Sci..

[41] Monteiro M., Newcombe V.F., Mathieu F., Adatia K., Kamnitsas K., Ferrante E., Das T., Whitehouse D., Rueckert D., Menon D.K. (2020). Multiclass semantic segmentation and quantification of traumatic brain injury lesions on head CT using deep learning: An algorithm development and multicentre validation study. Lancet Digit. Health.

[42] Kuo W., Häne C., Mukherjee P., Malik J., Yuh E.L. (2019). Expert-level detection of acute intracranial hemorrhage on head computed tomography using deep learning. Proc. Natl. Acad. Sci. USA.

[43] McLouth J., Elstrott S., Chaibi Y., Quenet S., Chang P.D., Chow D.S., Soun J.E. (2021). Validation of a deep learning tool in the detection of intracranial hemorrhage and large vessel occlusion. Front. Neurol..

